# Motivational Interviewing to Reduce Drug Use and HIV Incidence Among Young Men Who Have Sex With Men in Relationships and Are High Priority for Pre-Exposure Prophylaxis (Project PARTNER): Randomized Controlled Trial Protocol

**DOI:** 10.2196/13015

**Published:** 2019-07-04

**Authors:** Tyrel J Starks, Gabriel Robles, Mark Pawson, Ruben H Jimenez, Monica Gandhi, Jeffrey T Parsons, Brett M Millar

**Affiliations:** 1 Hunter College City University of New York New York, NY United States; 2 Doctoral Program in Health Psychology and Clinical Science Graduate Center City University of New York New York, NY United States; 3 Doctoral Program in Sociology Graduate Center City University of New York New York, NY United States; 4 Division of HIV, Infectious Diseases, and Global Medicine Department of Medicine University of California San Francisco San Francisco, CA United States

**Keywords:** HIV, pre-exposure prophylaxis, substance-related disorders, sexual behavior, sexual partners

## Abstract

**Background:**

Men who have sex with men (MSM) currently account for more than two-thirds of new HIV diagnoses in the United States and, among young MSM (YMSM) aged 20 to 29 years, as many as 79% to 84% of new infections occur between primary partners. Contributing to HIV risk, YMSM use drugs at comparatively high rates. To date, no interventions have been developed that specifically address the unique needs of partnered YMSM or incorporate a focus on relationship factors in addressing personal motivation for change.

**Objective:**

The study’s primary aim is to evaluate the efficacy of the PARTNER intervention and evaluate potential moderators or mediators of intervention effects. The study’s secondary aims were to gather ideographic data to inform a future effectiveness implementation study and develop a novel biomarker for pre-exposure prophylaxis (PrEP) adherence by analyzing PrEP drug levels in fingernails.

**Methods:**

PARTNER is a 4-session motivational interviewing–based intervention that integrates video-based communication training to address drug use and HIV prevention among partnered YMSM. This study utilizes a randomized controlled trial design to compare the PARTNER intervention with an attention-matched psychoeducation control arm that provides information about HIV-risk reduction, PrEP, and substance use. Participants are randomized in a 1-to-1 ratio stratified on age disparity between partners, racial composition of the couple, and relationship length. Follow-up assessments are conducted at 3-, 6-, 9-, and 12-months postbaseline. The study recruits and enrolls 240 partnered YMSM aged between 18 to 29 years at a research center in New York City. Participants will be HIV-negative and report recent (past 30-day) drug use and condomless anal sex with casual partners; a nonmonogamous primary partner (regardless of HIV status); or a serodiscordant primary partner (regardless of sexual agreement). Primary outcomes (drug use and HIV sexual transmission risk behavior) are assessed via a Timeline Follow-back interview. Biological markers of outcomes are collected for drug use (fingernail assay), sexual HIV transmission risk (rectal and urethral gonorrhea and chlamydia testing), and PrEP adherence (dried blood spots and fingernails for a novel PrEP drug level assay).

**Results:**

The study opened for enrollment in February 2018. Anticipated completion of enrollment is October 2021. Primary outcome analyses will begin after final follow-up completion.

**Conclusions:**

Existing research on partnered YMSM within the framework of Couples Interdependence Theory (CIT) has suggested that relationship factors (eg, dyadic functioning and sexual agreements) are meaningfully related to drug use and HIV transmission risk. Results pertaining to the efficacy of the proposed intervention and the identification of putative moderators and mediators will substantially inform the tailoring of interventions for YMSM in relationships and contribute to a growing body of relationship science focused on enhancing health outcomes.

**Trial Registration:**

ClinicalTrials.gov NCT03396367; https://clinicaltrials.gov/ct2/show/NCT03396367 (Archived by WebCite at http://www.webcitation.org/78ti7esTc.

**International Registered Report Identifier (IRRID):**

DERR1-10.2196/13015

## Introduction

### Background

Young men in same-sex relationships are confronted with a unique constellation of challenges specifically related to drug use and HIV risk. Men who have sex with men (MSM) accounted for 67% of all new HIV infections in the United States in 2016 and approximately two-thirds (64%) of those infections among MSM were observed in the youngest age cohorts (13 to 24 years and 25 to 34 years) [1]. Although rates remained largely stable among 13 to 24-year-old young men who have sex with men (YMSM), they rose by 21% among men aged 25 to 34 years [1]. Collectively, these data point to the early years of adulthood as a time of emerging and potentially escalating risk for HIV infection.

MSM in same-sex relationships are particularly vulnerable to HIV infection, with risk again concentrated in the early years of adulthood. Main partners accounted for 35% to 68% of new HIV infections among MSM [2,3]. Estimates suggest that rates of main partner transmission may be as high as 79% to 84% among YMSM aged 20 to 29 years [2], and this risk of main partner HIV transmission increases with discrepancy among the partners’ ages [2,3]. Sullivan et al [2] suggested that the increased risk for partnered men arises from the fact that men in relationships have sex more frequently with each other, with high rates of condomless anal sex (CAS).

The vulnerable age range defining YMSM corresponds largely to the developmental period of emerging adulthood. The transition from adolescence to adulthood has lengthened over time, with experts suggesting that emerging adulthood lasts from age 18 to 29 years with implications for both physical and mental health outcomes [4]. During this developmental period, emerging adults develop an increasingly firmer sense of sexual identity, solidify mechanisms to regulate emotions, and learn to develop romantic relationships [5-7]. Therefore, it is important to note that behaviors emerging adults adopt during this critical period not only affect current health but also have substantial implications for behaviors and health outcomes later in adulthood [5,7].

### Drug Use and Emerging Adult Young Men Who Have Sex With Men in Relationships: A Covariate of HIV Infection Risk

YMSM use drugs at higher rates than their heterosexual counterparts [8,9]. The most common drugs reported include cocaine, crystal meth, and other *party drugs* (eg, ketamine and gamma-hydroxybutyrate [GHB]) [10,11], as well as marijuana [12]. Drug use among YMSM is of particular concern because of its established association with HIV transmission risk behavior (ie, CAS with a partner of serodiscordant or unknown status) [13]. The fact that YMSM use drugs at higher rates than older MSM [14] and their heterosexual same-age counterparts [15,16] potentially compounds their risk for HIV infection.

A number of factors contribute to the need to develop tailored intervention strategies to address drug use among YMSM in relationships with other men. Unlike their heterosexual counterparts, being partnered is not associated with reductions in drug use among MSM [15]. Meanwhile, associations between drug use and HIV transmission risk behavior with casual partners remain significant among partnered MSM [17-19]. Sexual aspects of the relationship contextualize drug use for partnered men. Drug use covaries with sexual agreements [17,18], the understandings couples have about sex with partners outside their relationship [20]. Men in nonmonogamous relationships are significantly more likely to use drugs [17,18], and nonmonogamous relationships are characterized by more between-partner variability in use [17].

### Motivational Interviewing: A Basis for Intervention Development

Motivational interviewing (MI) is a client-centered approach to discussing a target issue: understanding that issue from a client’s perspective, enhancing individual motivation for change, and subsequently developing plans to achieve identified goals [21]. MI provides a framework for delivering information regarding the target behavior and specific strategies to cultivate personal motivation for change. It emphasizes the individual’s self-efficacy and autonomous capacity to make well-informed health decisions [21].

Brief MI-based interventions have shown efficacy in targeting substance use and sexual health among youth, including sexual minority youth [22-25]. Specifically, Parsons et al found empirical support for a 4-session MI-based intervention—termed the Young Men’s Health Project (YMHP)—to reduce both substance use and HIV transmission risk behavior among YMSM [25]. Secondary analyses of YMHP outcome data underscored the need to tailor interventions for YMSM in relationships. Starks and Parsons found that those YMSM who were partnered when they received YMHP showed essentially stable drug use over time, whereas a matched comparison sample of single men who also received the intervention showed significant and stable reductions in drug use [26]. Thus, although MI represents an adaptive and multipurpose platform with a proven record of success in integrated interventions focused on sexual health and substance use among YMSM, existing brief MI interventions may benefit from adaptations that specifically address relationship factors relevant to YMSM in relationships, particularly with regard to effects on drug use.

### Couples Interdependence Theory: Tailoring Motivational Interviewing for Partnered Young Men Who Have Sex With Men

CIT [27] has been applied to understand how relationship factors influence individual health outcomes for men in relationships with other men [28]. Through a process termed accommodation, partners within a relationship arrive at a shared goal or vision. When successful, the creation of the shared goal leads to a transformation of motivation, wherein partners consider the long-term effects of their decisions on their partner and their overall relationship [27,29-31]. This allows joint goals to draw on both individual-level and couple-level resources and increases the likelihood of goal accomplishment [30,31]. Notably, individual-level factors related to better dyadic functioning—including elements such as relationship satisfaction, commitment, communication, and relationship investment—facilitate the accommodation process [30].

CIT would suggest that an intervention tailored for partnered YMSM should incorporate components of relationship skill building. Male couples with better dyadic functioning will be better able to create shared sexual health goals and be more successful at the accomplishment of these shared goals once formed. However, dyadic interventions alone are insufficient to meet the diverse needs of partnered YMSM. For some couple-focused interventions, concurrent participation is required. This type of participation poses a logistical barrier for many couples as coordination of schedules is needed. Moreover, there is a possibility that one partner of the couple may be less able or motivated to participate. In addition, the implementation of couple-focused interventions may strain resources with providers as clinical skills and administrative-level concerns may arise [32]. Furthermore, researchers have indicated that participation in a couple-focused intervention may bias participation toward couples with higher relationship functioning [33,34].

### Biomedical Prevention for HIV Infection and Partnered Young Men Who Have Sex With Men

Daily use of Emtricitabine/Tenofovir Disoproxil Fumarate, FTC/TDF (Truvada), or PrEP reduces the risk of HIV infection substantially among those at risk. Recommendations from the US Centers for Disease Control and Prevention (CDC) [35] identify MSM in nonmonogamous relationships or relationships with an HIV-positive partner as high-priority candidates for PrEP. Despite this recommendation, research on gay male couples suggests that partnered YMSM face unique barriers to PrEP uptake.

Partnered men report being fearful that their primary partner may perceive them to be having sex with outside partners if they were to go on PrEP or discuss PrEP with their primary partner [36]. At the same time, the incorporation of HIV risk reduction plans (eg, PrEP use and consistent condom use) into sexual agreements can significantly reduce HIV transmission risk [37-39]; however, such inclusion requires the couple to be open to discussing PrEP. Gay men’s willingness to persuade a relationship partner to use PrEP was associated with their own willingness to take PrEP [38]. Collectively, these findings point to the need to facilitate communication between YMSM and their relationship partners about PrEP. Having an interventionist–a neutral party outside the relationship–introduce the topic may reduce anxiety related to initiating the conversation about PrEP and provide an opportunity for partners with some interest in PrEP to encourage less-interested partners to consider it.

### Novel Assessment of Pre-Exposure Prophylaxis Adherence

The extent to which PrEP achieves reductions in HIV risk is highly contingent upon adherence. Estimated reductions in infection risk are as high as 96% with full adherence to daily PrEP [40]. The assessment of adherence constitutes an inherent methodological challenge in PrEP studies. Behavioral measurements (eg, self-reports of missed doses and pill counting) are generally considered acceptable proxies for adherence but they can be influenced by participant biases such as social desirability or recall bias [41]. Testing for PrEP metabolites in dried blood spots (DBS) has emerged as a leading method for the objective assessment of adherence [42], although obtaining and storing high-quality samples is challenging [43]. Hair analysis can also be used to measure PrEP adherence [44-46]; however, the analysis requires a particular length of hair and therefore poses challenges for some participants–particularly males with very short hair [45].

Fingernail assays are a promising alternative approach to measuring PrEP adherence. Fingernail collection has high rates of acceptability and can be implemented with minimal training, with lower associated costs than blood collection. Fingernail assays have become increasingly used in the detection of illicit drug use [47-51]. Studies demonstrate that fingernail analysis may be more sensitive to detecting substances relative to hair assays [49,52,53]. Thus, developing a fingernail assay to measure Tenofovir (TFV) and FTC levels as biomarkers of adherence has potential utility for community-based and public health providers over DBS or hair adherence metrics in terms of acceptability and feasibility.

### Objective

The purpose of this study is to test the efficacy of a tailored MI-based intervention that specifically addresses factors relevant to drug use and HIV prevention for partnered YMSM who are high-priority candidates for PrEP. The study has 2 primary objectives. The first is to evaluate the efficacy of PARTNER with regard to 3 primary outcomes: (1) PrEP uptake and adherence, (2) sexual HIV transmission risk behavior, and (3) drug use. The second is to identify individual and relationship factors that moderate and/or mediate intervention effects. The study also has 2 secondary objectives: (1) gather ideographic data to inform a future effectiveness-implementation study and (2) validate the use of fingernail assays as a biological marker for PrEP adherence. In this study, the research team will collect both DBS and fingernail samples to assess the concurrent validity of PrEP drug or metabolite levels in each matrix.

The primary hypotheses are that YMSM receiving the PARTNER intervention will be more likely to initiate and/or be adherent to PrEP throughout the follow-up period than those in the education condition. It is hypothesized that they will have a lower probability of HIV transmission risk behavior at follow-up. In addition, it is hypothesized that the PARTNER intervention will be associated with lower levels of drug use (number of use days) at follow-up compared with the education condition. Secondarily, we hypothesize that levels of TFV/FTC observed in the fingernail assay will correlate highly with levels of TFV-diphosphate (TFV-DP) and FTC-triphosphate (FTC-TP) in DBS, providing evidence for the validity of this novel biological marker of adherence.

## Methods

### Trial Design

This study utilizes a randomized controlled trial design to evaluate the efficacy of the PARTNER intervention relative to an attention-matched psychoeducation control condition. Baseline assessment is conducted before randomization and receipt of intervention. Follow-up assessments are conducted at 3, 6, 9, and 12 months post baseline. Table 1 presents the study schedule for participation consistent with Standard Protocol Items: Recommendations for Interventional Trails guidelines (SPIRIT).

**Table 1:** Recommended content for the schedule of enrollment, interventions, and assessments.

Research activity		Study period					
		Enrollment	Allocation	Post-allocation			Close-out
Time point in months		0	0	3	6	9	12
**Enrollment**							
	Eligibility screen	X^a^	—^b^	—	—	—	—
	Informed consent	X	—	—	—	—	—
	Baseline	X	—	—	—	—	—
	Allocation	—	X	—			
**Interventions**							
	PARTNER	—	X	—	—	—	—
	Education	—	X	—	—	—	—
**Assessments**							
	Demographic; HIV status; pre-exposure prophylaxis uptake; Drug use; Relationship length;	X	—	—	—	—	—
	Drug Use Events; Positive drug assay; pre-exposure prophylaxis uptake/adherence, HIV transmission risk events; and Positive sexually transmitted infection test	X	—	X	X	X	X
	Dyadic functioning and sexual agreements	X	—	X	X	X	X

^a^Denotes that the research activity was conducted or data was collected at a particular time point.

^b^Indicates that the research activity was not conducted or data was not collected at a particular time point.

### Study Setting

All study assessments and intervention sessions are conducted at the Promoting Resilience, Intersectionality, Diversity, & Equity (PRIDE) Health Research Consortium affiliated with Hunter College of the City University of New York. PRIDE is located centrally in Manhattan with easy access to a mass public transportation hub linking it to the larger metropolitan area. Assessment and intervention sessions are conducted in private rooms.

### Eligibility Criteria

Participants must fulfill the following inclusion criteria to be enrolled in the study: (1) be aged 18 to 29 years, (2) have a main partner, for 1 month or longer, who is male and is aged 18 years or older, (3) be HIV negative (as confirmed by the rapid test), (4) have used drugs in the past 30 days, (5) have engaged in HIV transmission risk behavior in the past 90 days, (6) live in the New York City metropolitan area, and (7) be able to speak and read in English. Participants will be excluded from the study if they indicate any of the following: (1) any signs of serious mental illness or cognitive deficit and (2) history of intimate partner violence with their main partner.

### Interventions

#### PARTNER Intervention (Experimental Condition)

The PARTNER intervention comprises 4 sessions of MI to address 3 target behaviors that correspond to the study’s primary outcomes: drug use, PrEP uptake/adherence, and HIV transmission risk. Miller and Rollnick [54] suggested that 4 processes are ongoing during an MI session: engagement (establishment of a therapeutic alliance), focusing (clarification of session goals), evoking (eliciting speech in favor or change while softening arguments for the status quo), and planning (the identification of action steps that can be taken toward the accomplishment of an identified goal). The salience of these various processes is dependent upon the duration of the relationship between the interventionist and the participant as well as the client’s stage of change.

The first session emphasizes the engaging process. It begins with an introduction to the participant followed by an exploration of participant’s primary relationship, understanding how he and his partner handle sex outside their relationship, and enhancing motivation to reduce both drug use and HIV infection risk. This conversation then focuses on what strategies, if any, the participant and his partner use to manage their HIV risk. The interventionist then seeks to evoke motivation to reduce HIV related risk, potentially through PrEP uptake or adherence. The values card sort activity is utilized midway through the session to integrate a conversation about how the participant’s values are expressed in his relationship and the decisions made around HIV prevention. The second session engages the participant in a review of the previous week and then transitions to focus on drug use. The interventionist seeks to evoke motivation to reduce drug use, with particular attention given to how the participant’s relationship partner feels about use. The third session integrates a focus on the links between drugs and CAS with main and casual partners, PrEP, and presents video-based modeling to enhance communication skills. This facilitates a longer discussion about planning toward any identified goals. The interventionist gives particular attention to the role of relationship partners during the planning phase. The final session engages the participant in a review of the previous week. The session then proceeds to review the participant’s perception of the overall intervention process with emphasis on successes and challenges. The session emphasizes the planning process by inviting the participant to identify long-term goals related to the target behavior and develop plans to accomplish these goals. A discussion of relevant resources and referrals occurs during this time.

PARTNER incorporates a video-based approach to relationship skill building with a structured series of debriefing questions asked by the MI provider after the video is viewed. The video utilized was comprised 3 scenes, each depicting a different couple. It is integrated into Session 3 of the intervention and serves a dual purpose. First, it provides information specific to PrEP. Each scene depicts either a nonmonogamous or serodiscordant couple discussing PrEP and HIV prevention. In this way, participants viewing the video see men in relationships with men talking about reasons why PrEP might be relevant, while also receiving basic information about PrEP and its efficacy. Second, the videos directly teach communication skills through modeling. Each scene is divided into 2 parts. In the initial portion of the scene, the couple makes a specific communication error. Midway through the scene, a narrator interrupts the couple, explains their error, and suggests an alternative strategy. The scene then continues, and the couple communicates more effectively.

#### Education Intervention (Control Condition)

The education intervention is administered one-on-one by a health educator. The sessions are guided by a PowerPoint presentation that helps to insure fidelity of delivery while also increasing the participant’s engagement through the integration of pictures, animation, and video. The presentation is viewed on a personal computer located in the intervention room. Dual monitors are used to ensure that both the educator and participant can readily view material.

The condition comprises 4 sessions of health education addressing sexual risk and drug use. The sessions utilize a mixture of modalities including lecture, question and answer, and video. Session 1 focuses on HIV risk and prevention. Lecture content is supplemented by videos focusing on HIV transmission generally and HIV prevention strategies among gay and bisexual men specifically. Session 2 is focused on drug use. Information about the biological effects of drug use is largely provided through videos. Lecture content and discussion questions focus on the impact of drug use within the local gay community. Session 3 examines the intersection of drugs and sex in the local gay community. Education content also focuses on mitigating the risks associated with having sex while intoxicated. Session 4 again focuses on drug use with video and lecture-delivered content describing the signs and warnings of potential substance use disorders.

### Training of Interventionists

#### Experimental Condition

Initial intervention training includes a 2-day workshop for the interventionists employed by the study on MI delivered by a member of the Motivational Interviewing Network of Trainers (MINT) followed by a day long workshop on the structure of the PARTNER intervention. Ongoing coaching and supervision will occur, in addition to training on new interventions, as required. The training of the interventionists (experimental condition) is led by a clinical psychologist (principal investigator) who is a member of MINT and is from PRIDE. The training procedure consists of (1) a 2- to 3-month training period of role-play practice, coding and feedback, and supervision modeling, including mock sessions with *standardized participants* role-played by PRIDE research assistants; (2) weekly 1-hour supervision sessions with interventionists individually; (3) weekly group supervision with all the interventionists collectively; and (4) ongoing quality assurance and feedback using Motivational Interviewing Treatment Integrity (MITI) coding.

PARTNER will be delivered by postdoctoral fellows with formal training in mental health counseling, doctoral students in the Health Psychology and Clinical Science program at the City University of New York Graduate Center, and Masters level mental health clinicians employed at PRIDE. They will be trained in MI—and on the specific PARTNER protocol—by the principal investigator.

#### Education Condition

Training in the control condition (education intervention) is led by a postdoctoral fellow with extensive experience in the delivery of brief and structured intervention protocols. Educators delivering the education intervention will be project coordinators or graduate students with specific training in the delivery of the educational content related to drug use and sexual health. The training procedure consists of a 2- to 3-month training period of role-play practice, coding and feedback, and supervision modeling, including mock sessions with *standardized participants* role-played by research assistants.

### Fidelity Monitoring and Supervision

All PARTNER (experimental) and Education (control) sessions will be audio recorded. MI fidelity in PARTNER will be evaluated using the MITI coding system [55]. We will randomly select 25% of sessions by each interventionist to be coded. PRIDE maintains a team of trained MITI coders utilized in all of PRIDE’s National Institutes of Health-funded studies involving MI-based interventions. The educators will undergo similar fidelity procedures in which 25% of their education sessions will be evaluated for fidelity. These recordings will be reviewed and matched to a fidelity checklist that outlines the content of the Education session. Successful Education sessions will have accurately discussed at least 90% of the content.

### Outcomes

#### Drug Use Events

The number of drug use events is measured using a self-report interview commonly known as the timeline follow-back (TLFB [56]). Participants complete an interviewer administered 30-day TLFB interview of their drug and alcohol use [56]. Using a calendar, a research assistant coded whether any substance use occurred on a given day. On days when substance use occurred, the research assistant codes the presence of heavy drinking (ie, 5 or more alcoholic drinks) and/or the type of drug used (ie, marijuana, ketamine, methylenedioxy-methamphetamine (MDMA) or ecstasy, GHB, cocaine/crack, opiates or prescription drugs, or methamphetamine). In addition, this method will allow researchers to determine the number of days a particular drug was used and the total number of days in which any drug was used. Drug use is assessed at baseline, 3-, 6-, 9-, and 12-month follow-up appointments.

#### Positive Drug Assays

Positive drug tests via validated and commercially available fingernail assays will determine if a particular drug was used in the past 3 to 6 months. The assays are derived from a 5-panel detection system that detects the consumption of amphetamines/ecstasy/MDMA, cannabinoids, cocaine, opiates, and phencyclidine. The nail samples will be sent to the US Drug Testing Laboratories Inc for processing.

#### Pre-Exposure Prophylaxis Uptake/Adherence

PrEP uptake will be measured using self-report and PrEP adherence (among those on PrEP) and will be assessed using a TLFB interview approach analogous to that described earlier for drug use. Self-reported adherence will be verified by measuring PrEP metabolite concentrations in DBS via liquid chromatography/tandem mass spectrometry (LC-MS/MS). The results of the assays that analyze TFV-DP and FTC-TP in DBS quantify how many PrEP doses are taken over an average of 6 weeks as a metric of PrEP adherence. If participants did not report PrEP use at baseline, but report it at follow-up assessments, we will record their response to indicate that they initiated use and assess PrEP adherence via TLFB and DBS. With regard to the study’s secondary aim, PrEP adherence will also be assessed via nail samples. Levels of TFV-DP and FTC-TP observed in nail samples will be correlated with DBS results as a validation metric in accordance with the study’s secondary aim.

#### Number of HIV Transmission Risk Events

Self-report of CAS acts is measured using the TLFB. Using the TLFB calendar, the research assistant codes whether CAS occurred on a given day and how many times, and with what type of partner (main or casual and the partner’s HIV status). This method will allow researchers to determine the number of occurrences of CAS within the specified period. CAS is assessed at baseline, 3-, 6-, 9-, and 12-month follow-up appointments.

#### Positive Sexually Transmitted Infection Test

Positive sexually transmitted infection (STI) tests for urethral and rectal gonorrhea and chlamydia will be used as a supplemental proxy for HIV transmission risk behavior. STI tests used in addition to self-report have increasingly been used in studies focused on PrEP to measure CAS [57,58]. STI testing occurs at baseline, 3-, 6-, 9-, and 12-month follow-up appointments.

### Participant Timeline

See Table 1.

### Sample Size

We utilized the *Test for the Ratio of Two Negative Binomial Rates* module in PASS 19 [59] to evaluate power to detect between-condition differences in drug use frequency at any one follow-up time point. Power (1-beta) was set to .80 and the probability of type II error (alpha) was set to .05. The number of exposures was set to 30 (the number of days of use assessed in the TLFB). The rate of use in the education condition was set to 3, 6, and 9. Meanwhile dispersion values of 1, 2, and 4 were tested. Power was highest at low levels of dispersion and high base rates of use in the education condition. Under these circumstances, the study has power=.80 to detect a rate ratio of 0.70 or a 30% reduction in drug use instances. At high levels of dispersion and low base rates, the study would be expected to detect a rate ratio of 0.48.

Power to detect differences in HIV transmission risk behavior was calculated in 2 ways. First, using procedures similar to those described for drug use, the study has power to detect a rate ratio between 0.48 and 0.69 in the number of CAS events in the absence of PrEP. This calculation tested average rates of CAS in the absence of PrEP in the education condition of 1, 3, and 5 instances and dispersion was tested at values of 1, 2, and 4. Power to detect significant differences in the odds of a positive STI test was calculated using the *inequality tests for 2 proportions in a repeated measures design* module [59]. Analyses specified 4 waves of data, power (1-beta) was set to .80, and the probability of type II error (alpha) was set to .05. Autocorrelation (rho) was permitted to vary between .25 and .75. The proportion of positive STI diagnosis in the education condition was tested at values of .13, .10, and .07. Power declined as rho increased. Power increased with the proportion of positive diagnoses in the education condition. Analyses suggested that the study is adequately powered to detect large effects, associated with an odds ratio between 0.20 (under the least favorable conditions) and 0.32 (under the most favorable).

Power to detect differences in PrEP uptake was similarly calculated using the *inequality tests for 2 proportions in a repeated measures design* module [59]. Analyses specified 4 waves of data, power (1-beta) was set to .80, and the probability of type II error (alpha) was set to .05. Autocorrelation (rho) was permitted to vary between .25 and .75. We estimated that on average 35% of the education condition would be on PrEP during the follow-up period. The proposed sample is sufficient to detect an odds ratio of 1.90 even at the highest values of rho, which corresponds to approximately 51% of the PARTNER condition initiating PrEP. With regard to adherence, assuming approximately 100 participants (45% of the sample assuming 80% retention) are on PrEP at any follow-up point and 75% of the control condition is adherent to their PrEP medication (Cronbach alpha=.05; and rho=.25 to .50), results suggested that the study has power=.80 to detect an odds ratio of approximately 3.0.

### Recruitment

We will utilize a multifaceted recruitment effort including both active and passive approaches. Our previous research focused on MSM has indicated that Web-based recruitment is particularly efficient at reaching those who use substances and engage in sexual HIV transmission risk behavior [60,61]. We anticipate enrolling 6 new participants each month. Contacts for prescreened eligible participants obtained through the online screener will be contacted by email and phone and will be rescreened for project eligibility over the phone. If eligible, participants will have their baseline appointment scheduled and have their home-based survey emailed to them.

### Assignment of Interventions

#### Randomization

Participants will be randomly assigned using a stratified block randomization procedure using responses from the participant’s baseline questionnaire programed in Qualtrics. Specifically, randomization will account for: (1) age discrepancy: participant age difference with his partner (3 years or less/greater than 2 years); (2) relationship length with his main partner (2 years or less/greater than 2 years); (3) race/ethnicity makeup of the participant and his partner, for example, both partners identify as white and non-Hispanic/one or both partners identify as non-white or Hispanic. Thus, random assignment will occur only after participants have completed a baseline questionnaire. The random assignment will be performed by the Qualtrics system that has been programmed by the onsite data team. Participants will be randomized to 1 of 2 conditions, PARTNER (experimental condition) or education (control condition).

### Blinding

Study staff delivering the intervention and education conditions cannot be blinded to the condition they are delivering. To minimize contamination, these staff are cleared to only deliver one of the study conditions. Assessment staff are blinded to the condition at baseline as participants are not randomized until after the baseline assessment is completed. Assessment staff are not blinded to the condition at follow-up. Participants cannot be blinded to their assigned arm as participants will either be receiving an MI-based intervention in a modality analogous to psychotherapy or a control condition which is highly structured and educational in nature.

### Data Collection for Primary Outcomes

#### Dried Blood Spots Adherence Assays

Plasma levels of TFV only represent recent use, whereas PrEP drug or metabolite levels in DBS and hair represent long-term measures of adherence. DBS measurements of TFV-DP have proven useful in the evaluation of PrEP efficacy [62] and hair and DBS measures are highly correlated [63]. The DBS adherence assay allows for an estimate of the average number of PrEP doses taken using validated methods [64]. Blood samples for DBS preparation will be taken intravenously by a licensed phlebotomist.

#### Fingernail Assay for Tenofovir /Emtricitabine

The hair analytical laboratory (HAL) at the University of California San Francisco (UCSF) has developed expertise in the analysis of TFV/FTC concentrations in small hair samples using LC/MS-MS. The UCSF HAL will attempt to develop and validate a fingernail-based assay for PrEP adherence by measuring TFV/FTC levels in fingernails collected in the PARTNER study for the first time. Similar to the processing of hair samples, the fingernail samples will be pulverized using an Omni Bead Ruptor and weighed. TFV and FTC in the pulverized fingernail samples will then be extracted with 50% methanol/water containing 1% trifluoroacetic acid, .5% hydrazine dihydrochloride, and internal standard in a 37°C shaking water bath overnight (>12 hours) and then analyzed by LC-MS/MS. The DBS assays in the Antiviral Pharmacology Laboratory at the University of Colorado and the hair assays at UCSF are both peer validated and approved by the Division of AIDS Clinical Pharmacology and Quality Assurance (CPQA) program [65]; the fingernail assay, once developed and validated in year 1 of this proposal, will be similarly peer-reviewed by CPQA before testing during years 2 to 4. This specific fingernail assay will only be completed with fingernails collected from participants who report taking PrEP. Thus, a distinct fingernail drug assays will be analyzed with a separate set of fingernails collected from participants with the goal of measuring PrEP adherence. In addition, regardless of PrEP uptake, a nail sample will be collected from all participants for the purpose of drug testing.

#### Fingernail Assay for Drug Testing

Although studies have consistently supported the validity of self-reported drug use data [66-69], concurrent biological assessment enhances self-report accuracy [70]. Drug testing will be completed using the Nail Testing Panel from the US Drug Testing Lab. Participants will clip 100 mg of nail (approximately 10 clippings of 2 mm each). Clippings will be weighed on a jeweler’s scale to ensure the collection of an adequate sample. Clippings are transferred to a foil packet, stored at room temperature, and shipped to US Drug Testing Lab in a secure envelope. The assay detects amphetamines/MDMA, cannabinoids, cocaine, opiates, and phencyclidine use over a period of 3 to 6 months [71]. Results will be available in 5 to 7 business days [71].

#### Urethral Sexually Transmitted Infections

Urethral STIs (chlamydia and gonorrhea) are being tested with a kit from Identigene [72] and processed by Sunrise Laboratories. The kit uses the Gen-Probe Aptima Combo 2 Assay [73], which detects both chlamydia and gonorrhea. Participants will collect a urine sample and then place the specimen tube into a clear plastic biohazard bag.

#### Rectal Sexually Transmitted Infections

Participants will also perform a self-administered testing for rectal chlamydia and gonorrhea using a test kit from Sunrise Laboratories. The swab is approximately the size of a cotton swab and is grasped between the thumb and forefinger about an inch from the base. The swab is inserted until the fingers touch the anus and then it is rotated as it is removed. Swabs are stored in a specimen tube that is placed in a clear plastic biohazard bag. Specimens will be sent to Sunrise Labs who will provide us with test results, which we will then share with our participants. We will comply with New York City Health Department Reporting Requirements [74]. The purpose of STI testing is to provide a proxy assessment of HIV transmission risk behavior. We omit syphilis testing because it can be transmitted by a variety of behaviors, not all of which carry the risk of HIV infection.

#### HIV Testing

HIV testing will be conducted during the baseline assessment for those participants not on PrEP. Testing will be performed using Determine Ab/Ag 4th Generation Rapid HIV Test. A research assistant trained in couples HIV testing and counseling will utilize a lancet to collect a sample of blood from the participant’s finger. This drop of blood is then placed on the test paddle, and the paddle is placed in the test solution to culture. Test results are available in 20 min and delivered to participants immediately during the baseline assessment. For participants on PrEP, HIV testing occurs using an ARCHITECT HIV Ag/Ab Combo assay. This assay is a 2-step immunoassay to determine the presence of HIV p24 antigen and antibodies to HIV-1 (Group M and Group O) and HIV-2 in human serum and plasma using chemiluminescent microparticle immunoassay (Chemiflex) technology with flexible assay protocols. Blood samples are sent to and processed at Sunrise Laboratories.

### Data Management

Data will be collected onsite at baseline and at 3-, 6-, 9-, and 12-month follow-up periods. All survey instruments are administered using a Qualtrics-based computer-assisted survey instrument interface. To reduce the time required to attend the in-office baseline appointment, participants have the option of completing a portion of the baseline survey at-home Web-based before the appointment through a Qualtrics link. TLFB data are gathered by a trained interviewer using a data-entry system programmed in Microsoft Access (see below for additional details). The following sections also provide specific details related to the handling of biological specimens and related results. Study procedures have been reviewed by the Institutional Review Board of Hunter College. In addition, we have set up a Data Safety Monitoring Board consisting of leading experts in YMSM with particular specific expertise in randomized controlled trials, epidemiology statistical analysis, and clinical research broadly.

### Data Analysis Plan

Analyses will be conducted on both self-report and the biological metrics of adherence. Using self-reported data taken from TLFB interviews, we will utilize a latent growth curve (LGC) model to examine between treatment-group differences in PrEP uptake and adherence over the follow-up period. To do this, we will utilize a 0-inflated binomial distribution to model uptake (whether a participant is on PrEP) and adherence (whether the participant has maintained 4 or more doses of PrEP [75] weekly as prescribed) during each follow-up period. Separate growth processes (intercepts and slopes) will be specified for both the 0-inflated (PrEP uptake) and adherence portions of the model. Treatment condition will then be entered as a predictor of these growth factors. Separately, we will conduct analyses in which DBS data (estimated weekly doses) are used to determine dichotomous adherence to PrEP. Consistent with our secondary aim, exploratory analyses will compare results obtained using TFV/FTC levels assayed in fingernails to TFV-DP and FTC-TP concentrations measured in DBS via mixed-effects regression analysis calculated on log-transformed PrEP drug or metabolite concentration data from both matrices.

We define the occurrence of HIV transmission risk behavior using self-reported data as any CAS with a casual partner, serodiscordant main partner, or nonmonogamous main partner in the absence of adequate PrEP adherence (maintenance of at least 4 doses per week). Biologically, we will also consider a positive test result for either gonorrhea or chlamydia in the absence of PrEP adherence as an indicator of HIV transmission risk behavior. We will utilize an LGC model to examine between group differences on both of these 2 outcomes simultaneously. Mplus allows for the application of LGC modeling to dichotomous outcomes through the use of a log-link function. For each variable, a latent intercept and slope will be calculated. This will allow us to calculate the covariation between self-reported HIV transmission risk behavior and biological indicators of transmission risk over time. Treatment condition will then be entered into the model as a predictor of intercept and slope factors for each outcome.

Analyses will be conducted on both self-reported drug use instances (the total days of use reported for all drugs assessed on the TLFB, a count outcome) and drug use data from fingernail assays indicating the dichotomous occurrence of use during the assessment window. In this way, the rigorous biological measure of drug use is supplemented by self-reported data which provides information about a wider array of drugs and amount of use during the assessment period. Similar to the proposed analysis of HIV transmission risk behavior, we will model self-report and biological outcomes in the same LGC model. This will allow an examination of their covariation over time. Treatment condition will be entered into the model as a predictor of intercept and slope factors for each outcome.

### Data Monitoring

This current study protocol was approved by the City University of New York’s Human Research Protection Program (HRPP; Protocol Number 2017-0630) and is registered with Clinicaltrials.gov (NCT03396367). All participants undergo consent twice, first via an internet platform during the recruitment process, and second in-person at the baseline appointment

## Results

Recruitment was initiated in February 2018, and the first participant was enrolled on February 14, 2018. Enrollment is ongoing with the first 25% of the sample enrolled as of March 7, 2019. We anticipate completion of sample enrollment approximately in October 2021. Final follow-up assessments will be completed over the following year. Primary outcome analyses will commence therefore in October 2022 with the dissemination of findings anticipated over the following 6 months.

## Discussion

This study aims to address a critical gap in HIV prevention and drug use intervention options for partnered YMSM by developing an individually delivered intervention which incorporates a focus on relationship factors. The intervention therefore circumvents the demands of dyadic intervention delivery and is potentially useful with YMSM who are unable or unwilling to participate in couple-focused interventions but can still benefit from an intervention that addresses HIV and drug use within the context of romantic relationships.

Similar to existing dyadic interventions tailored for partnered YMSM, the PARTNER intervention incorporates a focus on communication skills training. The rationale for this focus is derived from CIT and premised on the assumption that enhancing communication skills will increase the likelihood that YMSM are able to engage with their partners in the formation and maintenance of HIV prevention and drug use goals. To minimize demands on interventionists while also facilitating individual delivery (which precludes a facilitated conversation between partners in session), the PARTNER intervention incorporates a novel video-based approach to communication skills training, which can be delivered by the MI providers with a minimum of additional training.

Finally, the proposed project tests an intervention aimed at addressing PrEP uptake in a high-priority population for whom no existing interventions are tailored (partnered YMSM meeting CDC criteria for PrEP candidacy). Furthermore, the secondary aims of this study include the development and validation of fingernail assays to assess PrEP adherence. This innovation has broad implications for the examination of PrEP adherence across research and service delivery settings.

The absence of data from relationship partners precludes the direct observation of dyadic influences on outcomes and potential cross-partner effects of the intervention. The use of individual (rather than dyadic) assessment was selected to enhance feasibility and is consistent with the focus on developing an individually delivered intervention for partnered men that removes the demands of dyadic participation. Participants will report relationship functioning and their perception of their partners’ drug use, sexual behavior, and PrEP uptake. This will provide proxy data that can inform future studies. In addition, generalizability is limited by a focus on cisgender MSM who are aged 18 to 29 years. This relatively narrow focus was chosen to reflect the epidemiology of a high-risk group and also to facilitate the tailoring of video-based modeling content. The sample is further limited to men living in the New York City metropolitan area.

## Abbreviations

CAS: condomless anal sex
CDC: Centers for Disease Control and Prevention
CIT: couples interdependence theory
CPQA: clinical pharmacology and quality assurance
DBS: dried blood spot
FTC: Emtricitabine
FTC-TP: FTC-triphosphate
GHB: gamma-hydroxybutyrate
HAL: hair analytical laboratory
LC-MS/MS: liquid chromatography/tandem mass spectrometry
LGC: latent growth curve
MDMA: methylenedioxy-methamphetamine
MI: motivational interviewing
MITI: motivational interviewing treatment integrity
MSM: men who have sex with men
PrEP: pre-exposure prophylaxis
PRIDE: Promoting Resilience, Intersectionality, Diversity, and Equity (PRIDE) Health Research Consortium
STI: sexually transmitted infection
TFV: Tenofovir
TLFB: timeline follow-back
UCSF: University of California San Francisco
YMHP: young men’s health project
YMSM: young men who have sex with men (aged 18-29 years, unless otherwise specified)
